# Germanane Quantum Dots Promote Metabolic Reprogramming of Immune Cells Toward Regulatory T Cells and Suppress Inflammation In Vitro and In Vivo

**DOI:** 10.1002/adfm.202521057

**Published:** 2026-01-18

**Authors:** Abhay Srivastava, Alireza Rafieerad, Weiang Yan, Keshav Narayan Alagarsamy, Qingdong Guan, Leena Regi Saleth, Michel Aliani, Sanjiv Dhingra

**Affiliations:** ^1^ Institute of Cardiovascular Sciences Department of Physiology and Pathophysiology Rady Faculty of Health Sciences St. Boniface Hospital Albrechtsen Research Centre Biomedical Engineering Program University of Manitoba Winnipeg Manitoba Canada; ^2^ Department of Immunology and Internal Medicine Manitoba Blood and Marrow Transplant Program CancerCare Manitoba University of Manitoba Winnipeg Canada; ^3^ Division of Neurodegenerative Disorders St. Boniface General Hospital Albrechtsen Research Centre University of Manitoba Winnipeg Canada

**Keywords:** immunomodulation, metabolic reprogramming, nanomaterials, quantum dots, regulatory T cells, sea‐horse analysis

## Abstract

Recently, nanomaterials have emerged as a tool in developing novel therapies against inflammatory diseases. Metabolic changes in immune cells direct the phenotype and function of the host immune system. Therefore, next‐generation immunomodulatory biomaterials should be designed to target metabolic pathways and trigger specific changes in immune cells to direct their fate toward an anti‐inflammatory phenotype. The current study reports the fabrication and first application of germanane quantum dots (GeHQDs) to modulate inflammation in cell culture and in vivo mouse model. Using rational design and synthesis strategies, our GeHQDs leverage the intrinsic anti‐inflammatory properties of germanane to provide a novel nanoplatform to trigger metabolic reprogramming of immune cells toward an anti‐inflammatory phenotype. These GeHQDs are spontaneously uptaken into the immune cells and trigger a switch in their phenotype toward regulatory T (Treg) cells. Metabolomic analysis suggested a downregulation in glycolytic flux and upregulation in fatty acid oxidation with an increase in mitochondrial respiration in the GeHQDs‐treated group, which is a typical signature of Treg cells. In an in vivo mouse model of systemic inflammation, GeHQDs treatment upregulated the circulating Treg cell number, improved the metabolomic profile and downregulated inflammation. The current study presents a new paradigm in targeting inflammatory diseases by modulating immune cell metabolism using next‐generation nanomaterials.

## Graphical abstract

Metabolic changes in immune cells direct the phenotype and function of the host immune system. Smart nanomaterials must target metabolic pathways to direct immune cell fate. This study reports the fabrication and first application of germanane quantum dots (GeHQDs) to modulate inflammation in vitro and in vivo. These GeHQDs leverage the intrinsic anti‐inflammatory properties to innovatively trigger metabolic reprogramming of regulatory T‐cells by regulating glycolytic flux and mitochondrial respiration to target inflammatory diseases.

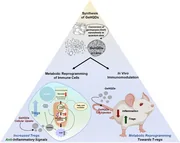

## 1 Introduction

Recently there has been growing interest in the development of nanomaterials‐based strategies to target the immune system and treat inflammatory diseases [1, 2, 3, 4]. Inflammation naturally occurs in response to tissue injury, infection, and or contact with the allergic substances in the body. This response of the host immune system is associated with a change in tissue microenvironment due to a dramatic metabolic shift within the host immune cells [5]. The metabolic changes in immune cells including alterations in glycolytic or mitochondrial oxidative phosphorylation (OXPHOS) pathways direct the phenotype and function of host immune cells and promote inflammation [6]. These changes in immune cell microenvironment are brought by several key metabolic regulators, enzymes, and immune‐metabolites [6, 7, 8, 9]. The immune cells responsible for inflammation such as effector T cells and neutrophils predominantly rely on glycolysis for energy production, proliferation, and biosynthesis of macromolecules. On the other hand, anti‐inflammatory immune cells responsible for promoting immune suppression such as regulatory T cells (Tregs) rely on OXPHOS, which is fueled by fatty acid β‐oxidation pathway. Therefore, in an inflammatory microenvironment these metabolic changes define cellular identity and modulate immune responses [6]. Hence, nanomaterials that can target metabolic pathways and trigger specific cellular metabolic changes within the immune cells will have the potential to provide therapeutic avenues against inflammatory and infectious diseases.

There are several reports that have shown promise in using nanomaterials in influencing immune cell function, thereby modulating the immune responses [10, 11, 12, 13, 14, 15, 16]. Also, some of the studies have reported that nanoparticles can alter metabolic environment of immune cells [15, 17, 18, 19]. However, there are very limited reports available on testing the efficacy of nanomaterials to regulate the phenotype and function of immune cells by influencing metabolism within the immune cells. The current study presents a new paradigm in the area of nanomaterials‐based biomedical applications where cell fate and function can be influenced by directing cellular metabolic shift. We synthesized germanane nanosheets‐derived quantum dots (GeHQDs) and evaluated their potential to modulate immune cell metabolism, phenotype and function. Recently, Xenes including germanene have been identified to have excellent biological and physicochemical properties, which are being exploited for their biomedical applications [20, 21]. In particular, hydrogenated germanene (GeH) flakes have been shown to possess enhanced bandgap and excellent optical properties, which facilitate their interaction with cells and foster potential applications of these materials for nanomedicine [22, 23, 24, 25]. Nonetheless, GeH flakes are air‐sensitive and oxidation can occur on the surface of these nanosheets under normal atmospheric conditions [26]. Furthermore, the crystal colloids of GeH nanosheets are not highly water‐dispersible resulting in prompt precipitation. These properties may affect their biomedical applications when material needs to be injected or circulated in the body. Another challenge with nanosheets is associated with their size and shape which decreases the cellular uptake and physical damage of these colloidal particles when they interact with biological environments. Therefore, such improvements in the physical properties of GeH, while maintaining its sophisticated optical and biological properties are undoubtedly beneficial for diverse immunoengineering applications and in other areas of nanomedicine. Hence, in the current study we reduced the size of GeH nanosheets to fabricate stable 0D quantum dots with enhanced biocompatibility and bioactivity over existing prototypes [27, 28]. We comprehensively characterized the physicochemical properties of GeHQDs and evaluated their anti‐inflammatory and immunosuppressive properties through in vitro cell culture model and in vivo lipopolysaccharide (LPS) induced inflammation model. Our data demonstrate that GeHQDs have the potential to reprogram metabolic pathways of immune cells under in vitro as well as in vivo conditions to induce Treg cell phenotype and suppress inflammation. We believe this study may open a new paradigm in developing therapeutic strategies against inflammatory diseases by targeting immune cell metabolism to modulate their function toward anti‐inflammatory phenotype and promote immune suppression.

## 2 Results

### 2.1 Preparation and Physicochemical Characterization of GeHQDs

**FIGURE 1:**
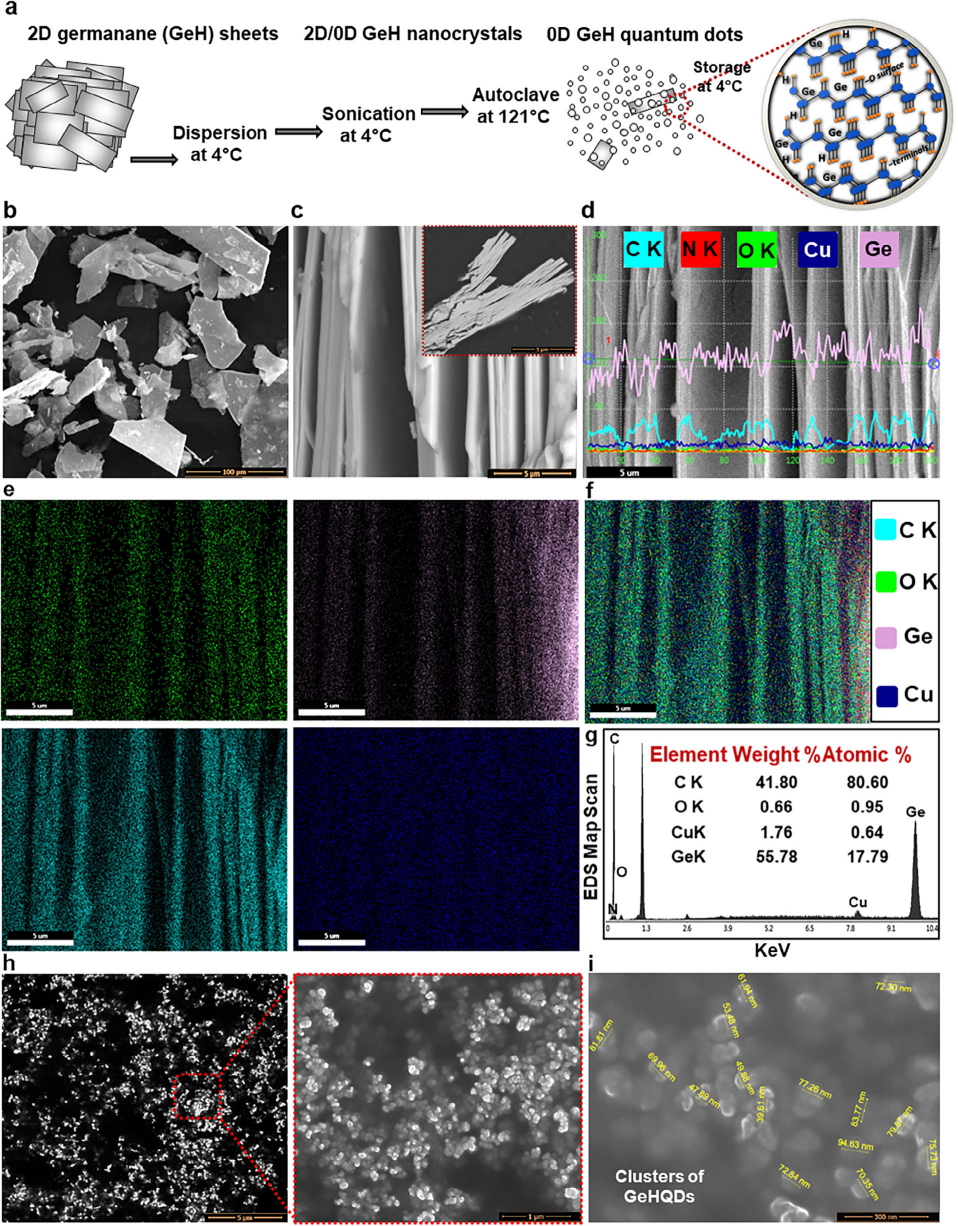
Schematic illustration of the synthesis and characterization of GeH nanosheets‐derived quantum dots. (a), The Proposed schematic model depicts the conversion of hydrogenated 2D germanane sheets to colloidal GeHQDs. (b), Morphological illustration of germanane flakes (scale bar 500 µm). (c), SEM cross‐sectional and backscattered images of GeH nanosheets. Our data revealed a comparative separation of the GeH layers without any significant impurity of elements with different atomic number, (scale bar 5 µm). (d), EDS line scan of germanane layers exhibits the elemental concentration and composition of these nanosheets. As shown here, germanium is the dominant element; a negligible intensity of oxygen further confirmed the purity of this material, (scale bar 5 µm). (e–g), EDS mapping and area scan analysis of germanane nanosheets was performed to quantify the weight and atomic percentage of different elements. As shown, the weight percentage of germanium is approximately 55%, (scale bar 5 µm). (h–i), SEM images of GeHQDs clusters at different magnifications ((h): scale bar 5 µm; (i): scale bar 300 nm).

Germanene is a buckled 2D van der Waals material with a crystalline hexagonal lattice structure [29]. Recently, several studies have reported that conversion of germanene to GeH nanosheets results in a tuned bandgap and significant electron mobility to the end product [22, 30, 31, 32]. The GeH contains an abundance of hydrogen groups which covalently bond to the germanium atoms in its structure [27, 28]. In the current study, we used commercially available flakes of GeH nanosheets and converted them to highly stable aqueous colloids of 0D GeHQDs. Briefly, the magnetic stirring and ultrasonic treatment were employed to the GeH solution in isopropyl alcohol to further expand the nanosheets. The resultant dispersions contained delaminated and oligo‐layers of GeH. Also, this treatment led to the conversion of GeH nanosheets into smaller pieces, as described in our recent work [33]. The decrease in dimensions of nanosheets to ultrasmall particles improves their stability and dispersibility. A schematic model depicting the transformation process and proposed molecular structure of GeH nanosheets‐derived quantum dots is presented in Figure 1a.

Next, we comprehensively characterized the microstructure and physicochemical properties of GeHQDs using scanning electron microscopy (SEM) and energy‐dispersive X‐ray spectroscopy (EDS) to confirm their successful synthesis. As shown in Figure 1b, the GeH flakes possess the specifications of a 2D monoelemental Xene with variable size fractions. Also, our SEM cross‐sectional image reveals a typical morphology of the GeH nanosheets with a well‐defined expansion of layers (Figure 1c and inset). Further, the backscattered SEM microgram of these nanosheets displayed a relatively homogenous characteristic of GeH without significant detection of irrelevant elements in its atomic composition. To further confirm this observation, we also examined the EDS line scan and area mapping for the chemical structure of this material (panel Figure 1d–g). These data confirm that germanium is the most dominant element in the chemical composition of the nanosheets. Further, the analysis of EDS measurements of GeH powder suggests that the weight and atomic percentage of germanium in this sample are 55.78% and 17.79%, respectively.

**FIGURE 2:**
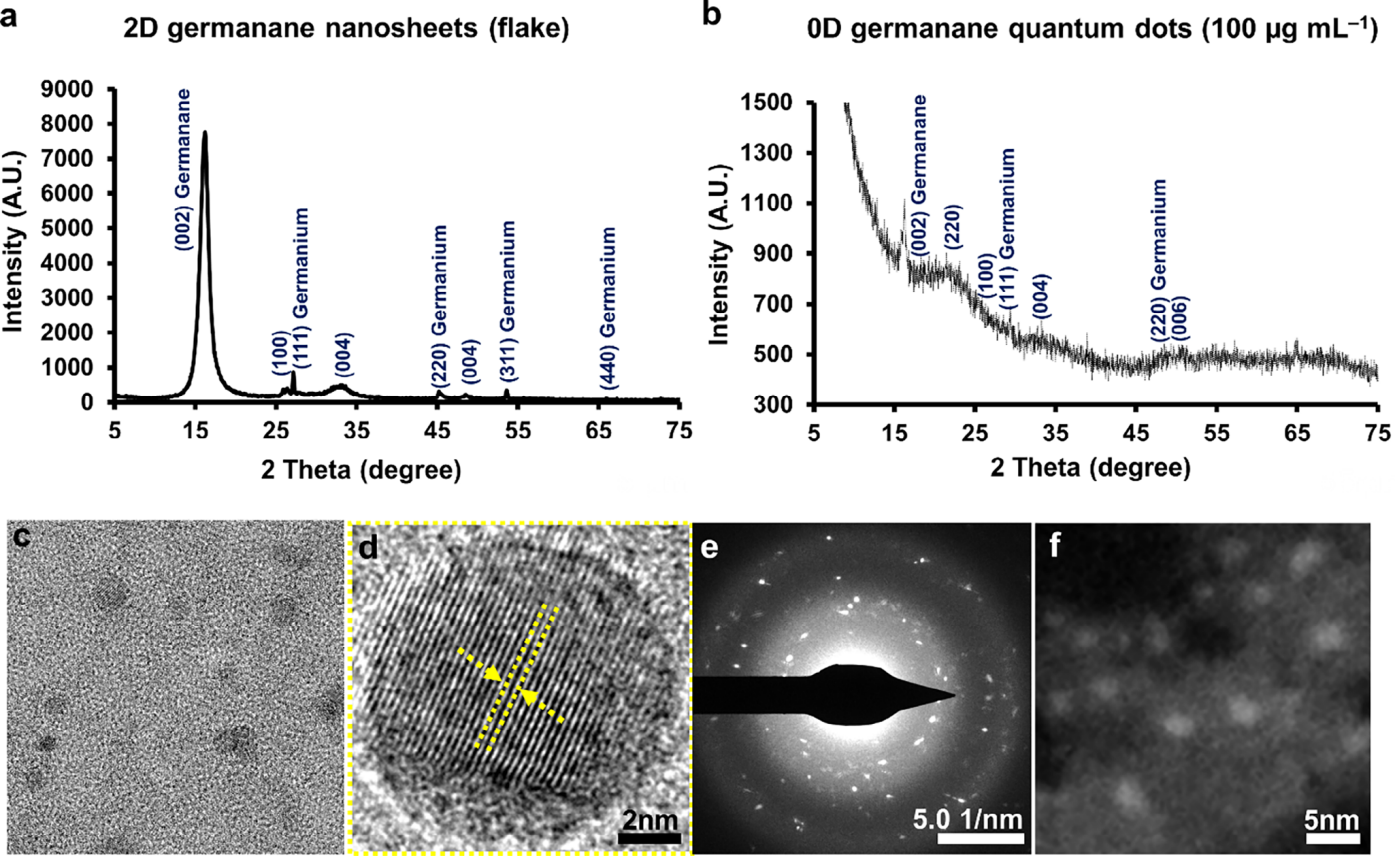
Phase and microstructural characterization of GeH quantum dots. (a,b), The XRD spectra display the phase pattern of GeH nanosheets and quantum dots, respectively. These data confirmed the relative characteristic peaks of germanium and hydrogenated GeH. The presence of (002) peaks at a 2‐theta of ≈ 16° in the XRD pattern of GeHQDs suggests the successful synthesis of GeH quantum dots without any significant phase change compared to GeH nanosheets. (c), TEM analysis showing distribution of 2D GeHQDs. (d), High‐resolution TEM image of GeH quantum dots. (e), The SAED pattern of as‐prepared GeHQDs clusters confirmed their crystalline structure. These data revealed the formation of GeHQD particles with an average size of 3–5 nm. (f), the scanning TEM images of these quantum dots clearly depict the presence of particles containing heavy germanium atoms.

Next, we characterized the microstructural properties, phase, and surface chemistry of GeHQDs using SEM, X‐ray diffraction (XRD), transmission electron microscopy (TEM), scanning TEM, selected area electron diffraction (SAED), and X‐ray photoelectron spectroscopy (XPS) analysis, as shown in Figure 1h,i. The SEM image of derived quantum dots showed a high‐yield production of uniformly nanosized GeHQDs and their clusters with an average diameter of around 68.75 nm. We also performed XRD characterization of germanane nanosheets and quantum dots (Figure 2a,b). Our data demonstrate that the XRD pattern of GeHQDs at 2‐theta range of 5–80 degree is almost similar to the parent material. Notably, a dominant peak at ≈16° corresponding to the (002) planes has been identified in the XRD spectra of germanane nanosheets, which concisely matches with the previous literature [30, 34]. Furthermore, the intensity of (111), (220), and (311) germanium peaks at 2θ of ≈ 28°, 46°, 54°, and 67°, respectively, was significantly downshifted in the XRD spectrum of GeHQDs. Additionally, our phase analysis of these particles revealed a minor (100) peak of germanium oxide (e.g., GeO_2_) at around 27°, indicating the purity of the end product. It is important to note that a negligible concentration of GeO_2_ (close to zero) in the XRD pattern of aqueous GeH quantum dots suspension suggests higher stability of GeHQDs compared to germanane nanosheets.

Besides, the TEM images of this material illustrated the formation of quantum dots, partially distributed into a germanene nanosheet (Figure 2c,d; Figure S1). The obtained 0D‐2D GeH crystals possess a lattice spacing of ∼2.2–2.4 Å and <2.0 Å, corresponding to the inner and outer planes of GeH nanosheets and quantum dots, respectively. In addition, the SAED pattern of GeHQDs exhibited a distribution of crystalline particles with random orientations (Figure 2e). Our scanning TEM (STEM) microgram clearly depicted a bright array of GeHQDs that further confirms the successful synthesis of particles (Figure 2f). Further, dynamic light scattering (DLS) analysis was performed to determine the particle size distribution of this material in aqueous dispersions of deionized water and cell‐culture medium (RPMI‐1640). The percentage distribution intensity (number‐weighted) was plotted (Figure S2) on the *y*‐axis against the particle diameter on the *x*‐axis. Our data suggest that the majority of particle clusters fall within the 100–250 nm size range, suggesting that agglomeration of these clusters in solution contributes to the observed size distribution in both deionized water and RPMI‐1640.

We also assessed the overall surface chemistry of GeH flakes and after converting them into GeHQDs by XPS wide scan spectra (Figure S3). The comparison of wide‐scan analysis of these samples revealed an efficient conversion of GeH flakes to quantum dots. The related germanium peaks [Ge 3d (∼26–38 eV), Ge 3p, Ge 2s, Ge LMM, and Ge 2p, corresponding to Ge and Ge─H bonding], carbon‐ and oxygen‐related peaks (C 1s and O 1s), which are attributed to the surface terminals of the materials and oxygen/CO_2_, and silicon (Si 2p), are dominant in these compositions, suggesting the purity and absence of irrelevant elemental compositions in the structure of GeHQDs. Additionally, the sharp O 1s peak at the binding energy of ∼530 eV partially refers to the oxygen‐containing groups available on the surface of these flakes and GeHQDs. The XPS narrow‐scan analysis of this material was reported in our previous work [33].

Moreover, the previous studies on optoelectronic applications of Xene and Xane have shown that these nanomaterials possess excellent optical properties [22, 35, 36, 37, 38]. In the current study we assessed the optical absorption pattern of GeHQDs to characterize the dispersibility and long‐term stability of these nanomaterials in aqueous media. The microscopic images of aqueous GeHQDs confirmed excellent dispersibility with no significant agglomeration of the particles after 90 days at 4°C (Figure S4). Also, the phase‐contrast and bright‐field micrographs revealed the stability of GeHQDs colloids at tested concentrations. Together, these data suggest successful preparation of stable GeHQDs with enhanced dispersibility.

### 2.2 Assessment of Biocompatibility of GeHQDs

Next, we assessed the biocompatibility of germanane quantum dots with mice spleen‐derived lymphocytes at two different doses of GeHQDs (2 and 100 µg/mL). The GeH quantum dots were incubated with lymphocytes for a period of 72 h, and lactate dehydrogenase (LDH) assay was performed in lymphocytes to assess cytotoxicity caused by GeHQDs. The biocompatibility of GeHQDs was also assessed by determining the viability of lymphocytes using Annexin V and PI staining. Our data demonstrate that GeHQDs were highly compatible with splenic lymphocytes as there was no significant difference observed in the LDH release and percentage of live cells in control and GeHQDs‐treated groups at either of the two doses. (Figure S5a,b).

### 2.3 Immunomodulatory Properties of GeHQDs

**FIGURE 3:**
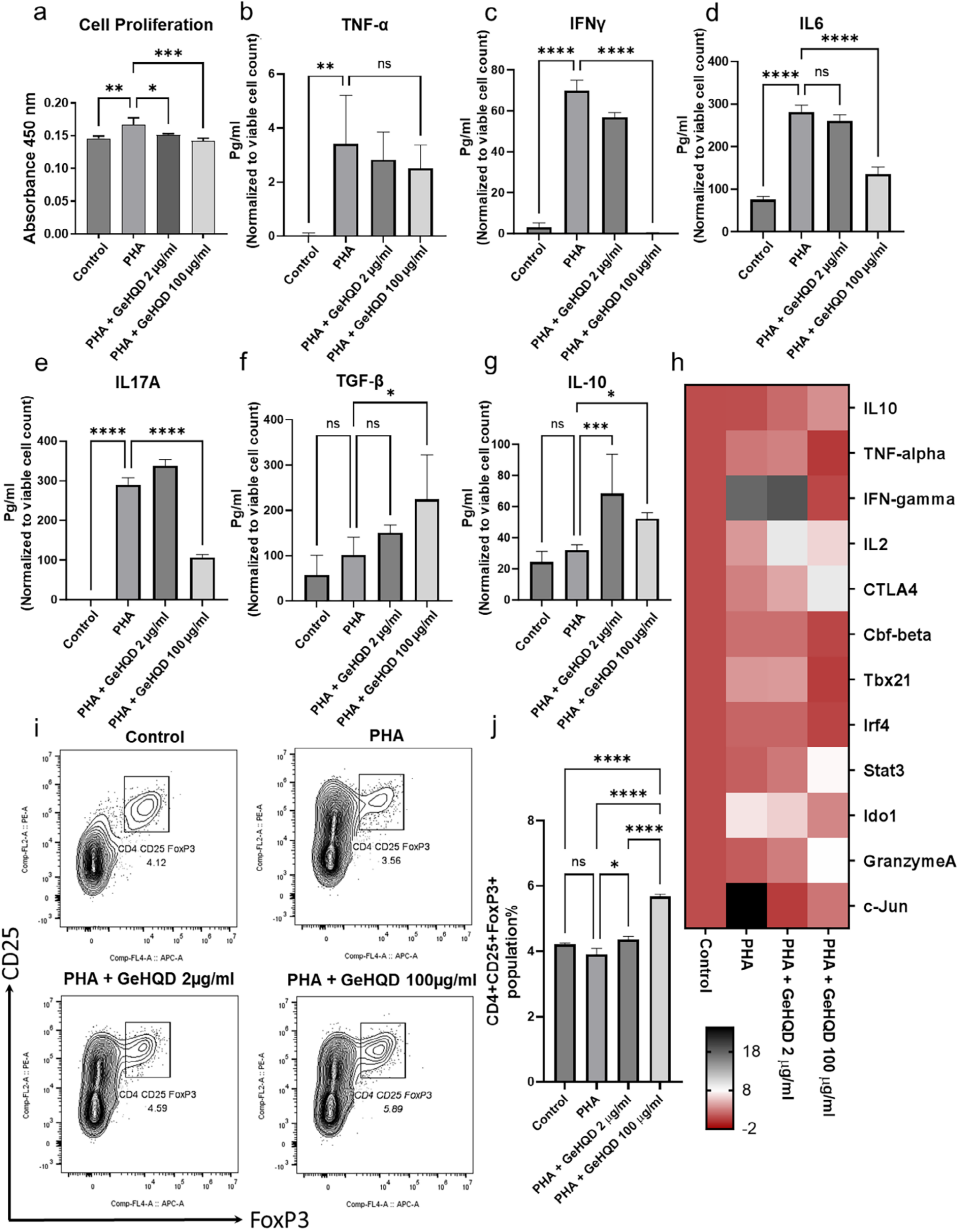
Immunomodulatory properties of GeHQDs: (a), Splenocyte proliferation was measured by WST proliferation assay. There was a decrease in the proliferation of activated splenocytes in the presence of GeHQDs. Four biological replicates were included per treatment group (b–g), Histograms show quantification of respective cytokines (TNF‐α, IFN gamma, IL‐6, IL‐17A, TGF‐β, and IL‐10,) in each group. Six biological replicates were included per treatment group. (h), The Heat map shows the quantification of respective genes in each group by PCR. Downregulated genes are in red and upregulated in white to grey. Four biological replicates were included per treatment group. (i), The panels depict Treg cell population percentage for respective groups by flow cytometry. The *x* axis is FoxP3 positive cells and *y* axis denotes CD25 positive cells. (j), Quantification of CD4^+^CD25^+^FoxP3^+^ Treg cell population in each group. Five biological replicates were included per treatment group for flow cytometry. All histograms are plotted as mean±SD. P‐values were calculated using one‐way ANOVA with Tukey's honest multiple comparison test, ^*^ *p* < 0.05, ^**^ *p* < 0.01, ^***^ *p* < 0.001, ^****^ *p* < 0.0001 and ns for *p* > 0.05.

To study the immunomodulatory properties of GeH quantum dots, splenocytes were treated with phytohemagglutinin (PHA) to activate them and mimic inflammatory conditions. The PHA‐activated splenocytes created a proinflammatory environment, as we observed higher proliferation of cells [39, 40], and treatment with GeHQDs downregulated the rate of splenocyte proliferation (Figure 3a; Figure S6a–d). We also measured cytokine levels in the cell culture supernatant and found a significant increase in the levels of proinflammatory cytokines TNF‐α, IFNγ, IL6, and IL17A in response to PHA exposure, and GeHQDs treatment decreased the levels of these cytokines in activated splenocytes (Figure 3b–e). Also, there was a significant increase in anti‐inflammatory cytokines including TGF‐β and IL‐10, after treatment with GeHQDs (Figure 3f,g; Figure S7). These data strongly suggest a shift from pro‐inflammatory to anti‐inflammatory environment after treatment with GeHQDs.

To understand the phenotypic and functional changes in splenocytes in response to GeHQDs treatment, we performed further analysis and observed that GeHQDs significantly downregulated the mRNA levels of transcription factors, C‐Jun, IRF4, IDO1, TBX21, and CBF‐β required for proinflammatory cellular phenotypes (Figure 3h; Figure S7). These findings further confirm that treatment with GeHQDs changed the phenotype of splenocytes from pro‐inflammatory to an anti‐inflammatory phenotype. We also observed that GeHQDs treatment upregulated STAT3, CTLA4, IL‐2, and Granzymes in activated splenocytes, which are known for anti‐inflammatory action in regulatory T (Tregs) cells (Figure 3h; Figure S7). The Treg cells are a subset of immune cells that help in suppressing inflammatory responses by downregulating the proliferation of effector T cells [41]. We performed further analysis to explore if there was a shift in the phenotype of T cells toward Treg cells, our flow cytometry data demonstrate that GeH quantum dots were able to significantly increase the number of CD_4_^+^CD_25_^+^FoxP_3_^+^ Tregs population in mice splenocytes [42] (Figure 3i,j; Figures S8 and S9). These data indicate that GeHQDs treatment promotes immune suppression by inducing Treg cell phenotype.

### 2.4 GeHQDs Treatment Shifts Cellular Metabolism Toward Treg Phenotype

**FIGURE 4:**
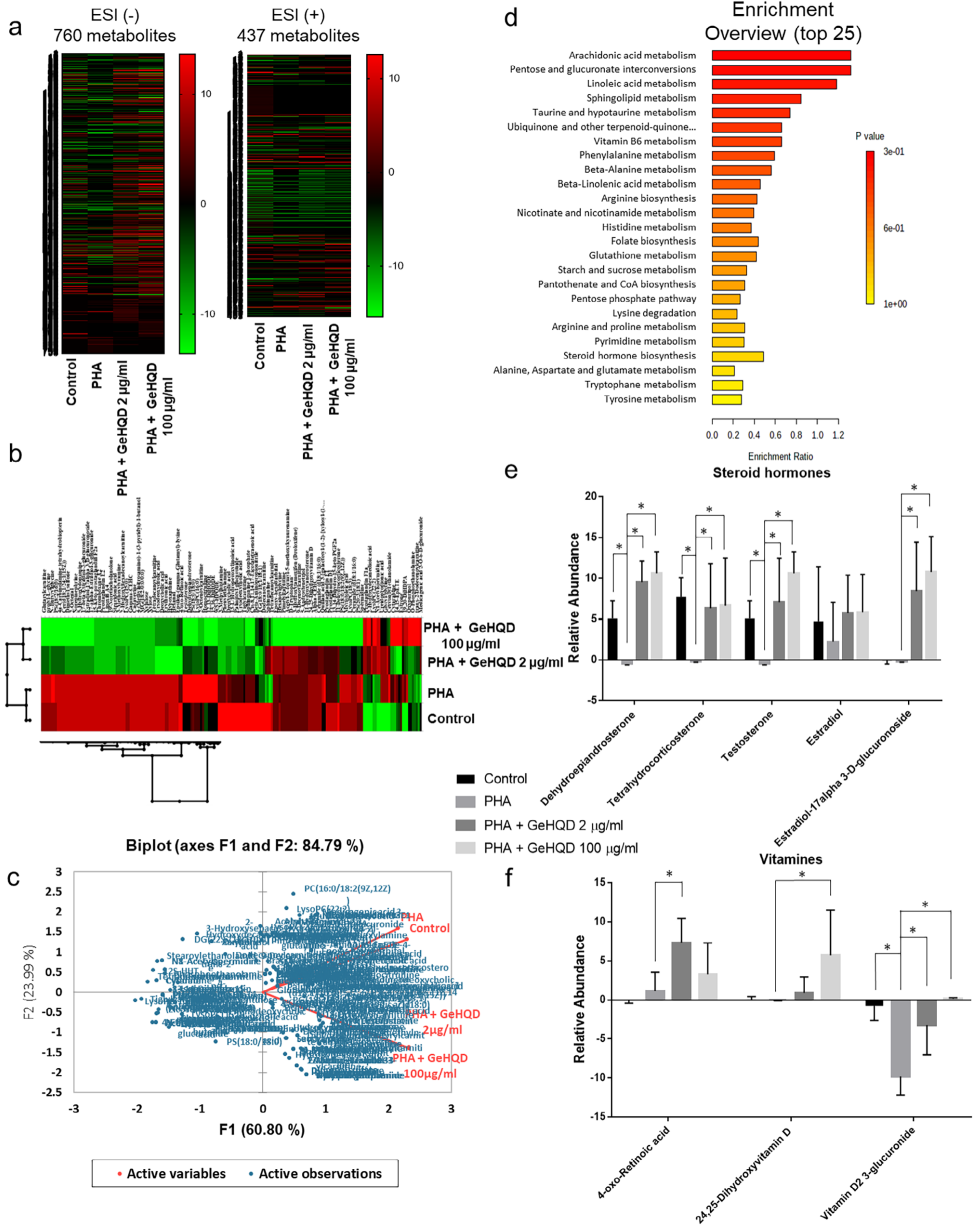
GeHQDs treatment alters immune cell metabolism: (a), Heat map showing global metabolic profile of individual metabolites detected in both ESI negative and positive modes in different groups. (b), The panel shows a hierarchical heat map of metabolomic analysis of all the significantly changing metabolites, where a shift in the metabolic profile of cells can be observed among different groups. (c), The Figure demonstrates a metabolic shift between the control, PHA treated immune cells and GeHQDs‐treated cells in a principal component analysis biplot of the significantly affected metabolites. (d), Enrichment analysis for significantly affected metabolites between the PHA and GeHQD‐treated groups. (e,f), Histograms depicting comparative abundance of steroid hormones and vitamins within different groups and quantification of respective cytokines in each group. Four biological replicates were included per treatment group. The histograms are mean±SD. P‐values were calculated using two‐way ANOVA with Tukey's multiple comparisons performed for two variables, ^*^ *p* < 0.05.

Next, we wanted to investigate the mechanisms of GeH quantum dots‐mediated induction of Treg cell population and suppression of immune responses. It is known that metabolic changes in immune cells play an essential role in directing the cell fate and function [43, 44]. Hence, in order to comprehend these changes, we performed global metabolomic analysis (using LC‐QTOF/MS) in activated immune cells treated with GeHQDs (Figure 4a). There was a significant difference observed in the metabolic profile of splenocytes after activation and following GeHQDs treatment, in fact we found a switch in the metabolic signature between control, activated immune cells, and GeHQDs‐treated cells (Figure 4b,c). Specifically, there were significant alterations observed in metabolites associated with carbohydrate, lipid, amino acid, and nucleic acid metabolism (Figure 4d), which was further confirmed by pathway analysis (Figure S10a,b). Interestingly, within the altered parameters we recognized certain key metabolites that promote the phenotype and function of Treg cells. There was a significant increase in the lipid and fatty acid metabolism in GeHQDs‐treated immune cells in comparison to PHA activated cells (Figure S10a,b). The upregulation in fatty acid metabolism is a strong indication for increased mitochondrial oxidative phosphorylation (OXPHOs), which is preferred by Treg cells [6, 41, 44]. We also detected higher abundance of steroid hormones including dehydroepiandrosterone, tetrahydrocorticosterone, testosterone, estradiol and estradiol‐17α3‐D‐glucuronoside in GeHQDs‐treated cells compared to activated immune cells. Similarly, the levels of vitamins including 4‐oxo‐retinoic acid, 24,25‐dihydroxy vitamin D, vitamin D2 3‐glucuronide, vitamin K 1 2,3‐epoxide, and pyridoxine were significantly higher in GeHQDs‐treated cells compared to activated immune cells (Figure 4e,f). All these metabolic changes are typically the indicators of a shift toward Treg cell phenotype. Therefore, these data strongly suggest that treatment of mixed population of immune cells with GeHQDs promotes a switch toward Treg phenotype [41, 44].

**FIGURE 5:**
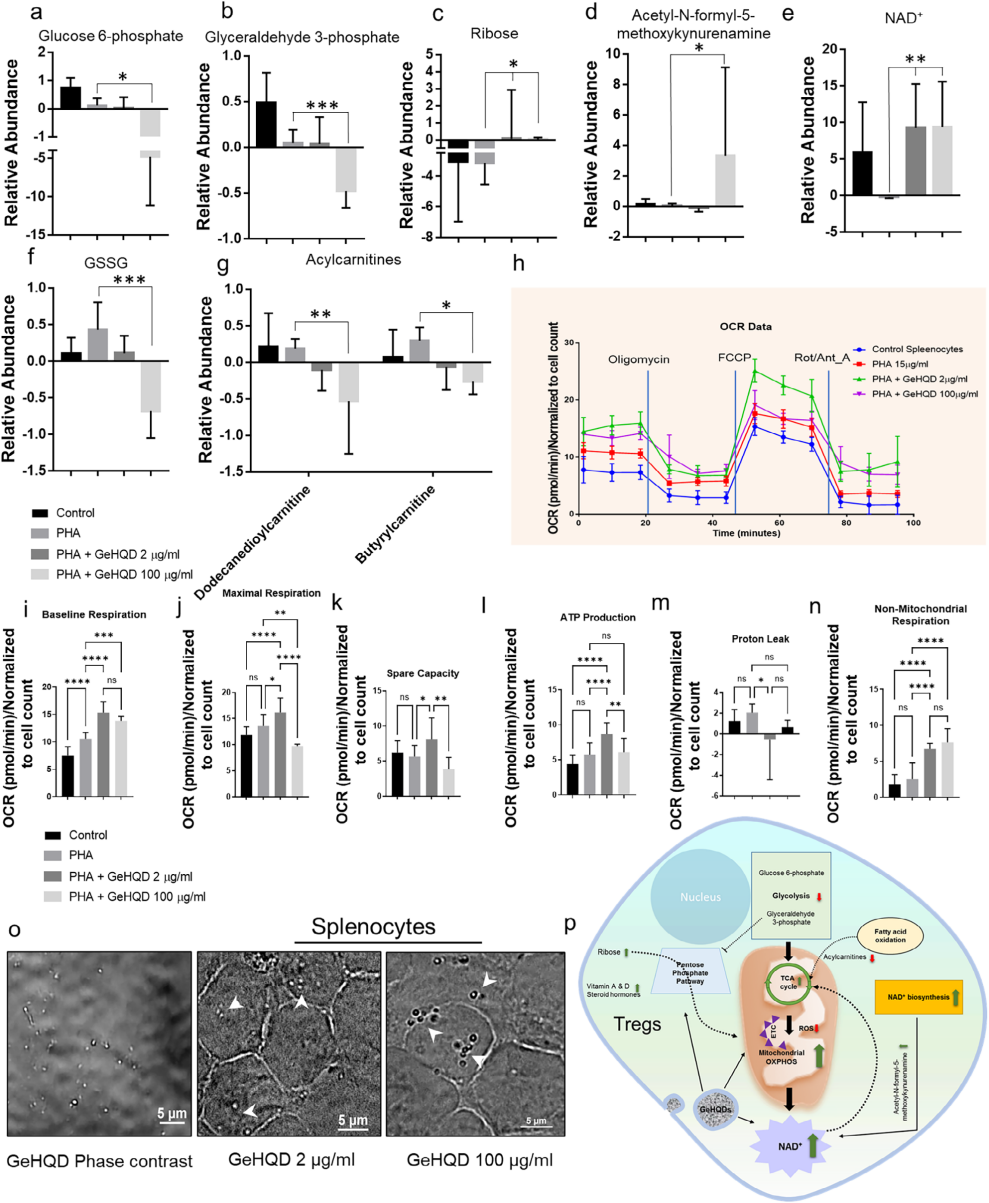
GeHQDs triggers cellular metabolic shift and improves mitochondrial function stimulating Treg phenotype and function: (a–g), Histograms showing comparative abundance of glucose‐6‐phosphate, glyceraldehyde‐3‐phosphate, ribose, acetyl‐N‐formyl‐5‐methoxykynurenamine, NAD^+^, oxidized glutathione and acylcarnitines respectively; Four biological replicates were included per treatment group. (h), The panel shows oxygen consumption rate in response to specific inhibitors, and measures mitochondrial respiration. (i–n), Histograms show baseline respiration, maximal respiration, spare respiration capacity, proton leak, ATP production and non‐mitochondrial respiration respectively. Five biological replicates were included per treatment group. (o), Phase contrast images showing GeHQDs (first panel), uptake of GeHQDs by splenocytes (second and third panel) at two different concentrations 2 and 100 µg/mL respectively. (p), Schematic of metabolic changes in response to GeHQDs favouring Treg cell phenotype and function. The histograms are mean±SD. P‐values were calculated using Two‐way ANOVA with Tukey's multiple comparisons performed for two variables, ^*^ *p* < 0.05. ^**^ *p* < 0.01, ^***^ *p* < 0.001, and ns *p* > 0.05.

Our data further suggest that the phenotypic and functional changes due to GeHQDs treatment facilitated the formation of a cellular environment favourable for increased mitochondrial oxidative phosphorylation and decreased glycolysis. This is a typical signature of a switch toward the Treg phenotype. We observed reduced abundance of key glycolytic metabolites including glucose‐6‐phosphate and glyceraldehyde‐3‐phosphate, suggesting a reduced activity of the glycolytic pathway. One of the most affected metabolites in activated immune cells was nicotinamide adenine dinucleotide (NAD^+^). The NAD^+^ is an essential metabolite that is needed by the cells for biochemical homeostasis, normal mitochondrial activity and scavenging cellular reactive oxygen species (ROS) [45]. We found a significant decrease in NAD^+^ abundance in PHA activated immune cells compared to the control group, treatment with GeHQDs prevented this decrease. Further, we also observed an increase in the abundance of ribose that bypasses the pentose phosphate pathway and increases mitochondrial activity and acetyl‐N‐formyl‐5‐methoxykynurenamine levels, which is formed from tryptophan breakdown assisting in NAD^+^ biosynthesis. Also, there was a decline in the levels of acylcarnitine metabolites such as dodecanedioylcarnitine and butyrylcarnitine implying increase in fatty acid oxidation in response to GeHQDs treatment which would further increase mitochondrial OXPHOS. (Figure 5a–g). Therefore, treatment with GeHQDs increases mitochondrial activity in immune cells. Hence, in the next experiments we performed Seahorse analysis to understand the effect of GeHQDs treatment on mitochondrial respiration in immune cells. Our data demonstrate that GeHQDs exposure improved overall mitochondrial respiration as well as ATP production and reduced proton leak in the cells (Figure 5h–n). The improved mitochondrial respiration and ATP production are essential for the immunosuppressive action of Treg cells [41, 44].

Interestingly, in our studies we also observed that GeH quantum dots were able to enter the splenocytes (Figure 5o). Some of the previous studies have reported that quantum dots are readily uptaken by cells and influence the intracellular pathways [46]. Therefore, in the current study, GeHQDs get internalized into splenocytes and create a unique biochemical flux, that triggers metabolic changes in the cells and stimulates phenotype change toward Treg cells. A schematic description of these events is presented in Figure 5p.

### 2.5 GeHQDs Induced Tregs Influence Key Immune Cell Interactions Toward an Anti‐Inflammatory Environment

**FIGURE 6:**
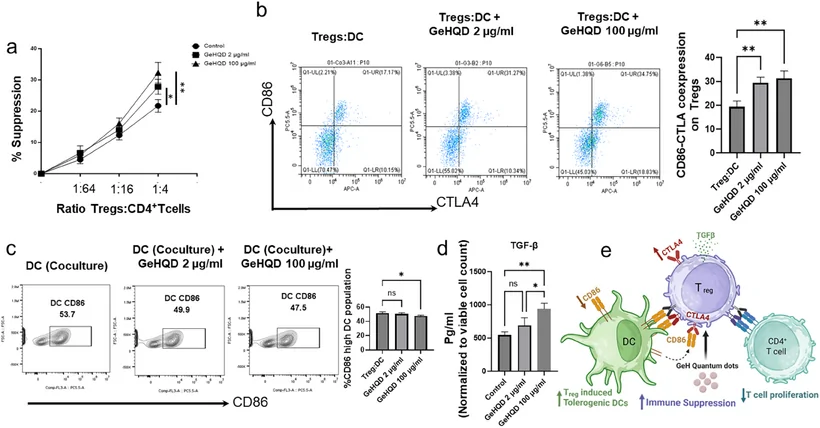
Effect of GeHQDs on immune cell interactions: (a), Graph showing suppressive effect of GeHQDs induced Tregs on CD4^+^ T cells. (b), Flow cytometry analysis of Treg cells for CTLA4 and CD86. (c), Flow cytometry analysis showing a decrease in CD86^+^ DCs in the presence of GeHQDs‐induced Tregs. (d), Histogram showing quantification of TGF‐β secretion during Treg and DC cell interaction in the presence of GeHQDs. (e), Schematic representation showing mechanistic insight on how GeHQDs‐induced Tregs can modulate immune cell function by regulating DCs and CD4^+^ T cells respectively. Four biological replicates were included per treatment group. The histograms are mean±SD. P‐values were calculated using One‐way ANOVA with Tukey's honest multiple comparison test, ^*^ *p* < 0.05. ^**^ *p* < 0.01, ^***^ *p* < 0.001.

As described in the above section, exposure to GeHQDs shifts the metabolism of splenocytes toward the Treg phenotype. Next we wanted to investigate how induced Treg (iTreg) cells influence other immune cells to create an anti‐inflammatory and immunosuppressive environment. The Treg‐mediated suppressive mechanisms include interaction with CD4^+^ T cells and antigen‐presenting cells (APCs). Hence, we studied interactions of iTregs with these two cell types. We observed that CD4^+^ T cells were suppressed significantly when cocultured with GeHQDs‐induced Tregs. The suppression is increased as the number of iTreg cells increases (Figure 6a; Figure S11a–d). The Treg cells promote immune‐suppression by upregulating the induction of tolerogenic APCs/dentritic cells (DCs), tolerogenic DCs are known to prevent activation of CD4^+^ T cells [47]. During interaction between Tregs and DCs, CTL‐associated protein‐4 (CTLA4) on the surface of Tregs binds with CD86 on DCs surface and removes it from DCs, thereby converting them into a tolerogenic phenotype [48]. These CD86‐deficient DCs trigger cell death, anergy or a state of dormancy in the CD4^+^ T cells [47]. We co‐cultured GeHQDs‐induced Tregs with DCs and found a significant increase in CTLA4^+^CD86^+^ Treg population, a subsequent decrease in the expression of CD86 on the surface of DCs and increased secretion of TGF‐β in the presence of GeHQDs (Figure 6b–d; Figure S12). To further validate our claims, we examined the effect of treatment with GeHQDs on naïve CD4^+^ T cells, and observed a significant increase in CD4^+^CD25^+^FoxP3^+^ regulatory T cells along with increased cell proliferation and mitochondrial membrane potential. Further, secreted TGF‐β levels in response to GeHQDs treatment were significantly elevated suggesting that GeHQDs trigger a favourable environment for conversion of naïve CD4^+^ T cells to a regulatory T cells phenotype. There was no change in TNF‐α levels suggesting that GeHQDs promoted an anti‐inflammatory environment (Figure S13a–h). Hence, in the current study GeHQDs‐induced Tregs modulate both CD4^+^ T cells and APCs to create an immune‐suppressive, anti‐inflammatory environment. These findings provide a mechanistic insight on how GeHQDs regulate immune cell function (schematic in Figure 6e).

### 2.6 GeHQDs Treatment Promotes Treg Cell Induction and Downregulates Inflammation in Vivo in a Mouse Model

**FIGURE 7:**
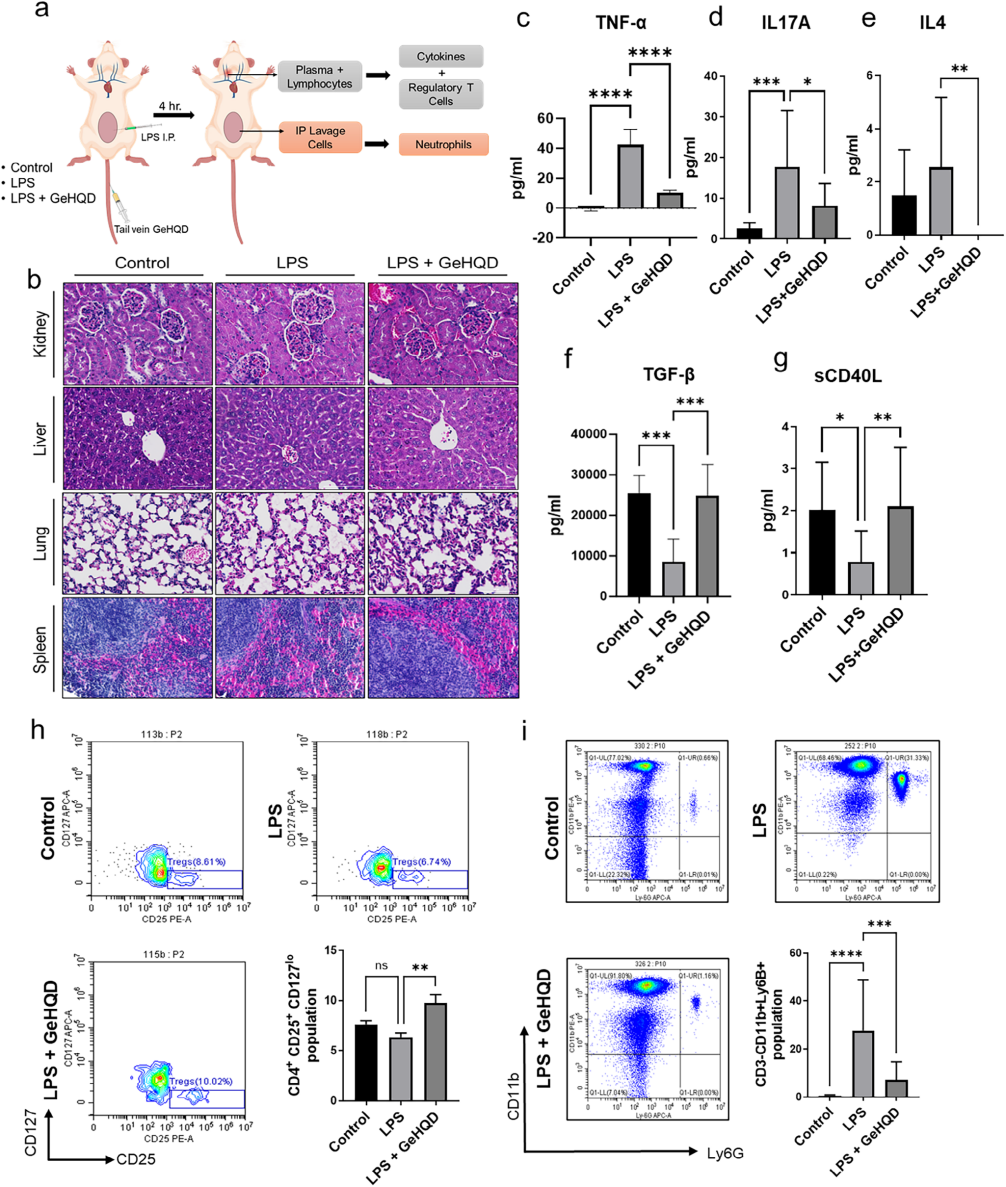
GeHQDs treatment modulates inflammation in a mouse model in vivo. (a), The Figure depicts the in vivo experimental design: CD1 mice were treated with LPS to induce inflammation. (b), H & E staining of the kidney, liver, lungs, and spleen showed no acute toxicity in these organs. Six biological replicates were included per treatment group. (c–g), Quantification of cytokines (TNF‐α, IL17A, IL4, TGF‐β, and sCD40L) in mice plasma from different groups by ELISA and Legendplex flow‐based cytokine array. Five biological replicates were included per treatment group. (h), CD4^+^CD25^+^CD127^lo^ Tregs cell population was analyzed in mice blood from different groups by flow cytometry. (i), CD3^−^CD11b^+^Ly6G^+^ neutrophils were analysed in peritoneal lavage using flow cytometry. Eight biological replicates were included per treatment group. The histograms are mean±SD. P‐values were calculated using One‐way ANOVA with Tukey's honest multiple comparison test, ^*^ *p* < 0.05. ^**^ *p* < 0.01, ^***^ *p* < 0.001.

Next, we validated our in vitro findings in an in vivo mouse model of lipopolysaccharide (LPS) induced inflammation. A schematic of the in vivo experimental design is presented in Figure 7a. To induce inflammation, male CD‐1 mice (6–8 weeks old) were injected with LPS (1 mg/kg body wt.) intraperitoneally. First, we performed in vivo biodistribution and biocompatibility assessment of GeHQDs. After an intravenous injection in mice the GeHQDs were abundantly present in the hepatic blood vessels, liver, lung and kidney tissues (Figure S14). Further, our data demonstrate that administration of GeHQDs at a dose of 2 mg/kg body wt., did not cause any toxicity in mice in vivo, as there were no histopathological changes observed in vital organs (kidney, liver, lung, and spleen) after injection of quantum dots (Figure 7b), which was also reflected in physical appearance scoring data showing improvement in mice treated with GeHQDs when compared with the LPS group (Figure S15).

To assess the anti‐inflammatory and immunosuppressive effects of GeHQDs in vivo, we performed several assays. The LPS injection in mice led to an increase in the levels of pro‐inflammatory cytokines including TNF‐α, IL17A, and IL4 in the blood, and treatment with GeHQDs significantly decreased these cytokines (Figure 7c–e). Furthermore, GeHQDs prevented LPS‐induced decrease in anti‐inflammatory cytokines TGF‐β and sCD40L (Figure 7f,g). We also found a significant increase in the number of CD4^+^CD25^+^CD127^lo^ Treg cells in GeHQDs‐treated mice (Figure 7h; Figures S16 and S18). Interestingly, GeHQDs treatment also mitigated LPS‐induced increase in the infiltration of CD11b^+^Ly6G^+^ neutrophils in the peritoneal cavity of mice (Figure 7i; Figures S17 and S18). These data confirmed that GeHQDs‐treatment has the potential to promote immune suppression and modulate inflammation under in vivo conditions as well.

**FIGURE 8:**
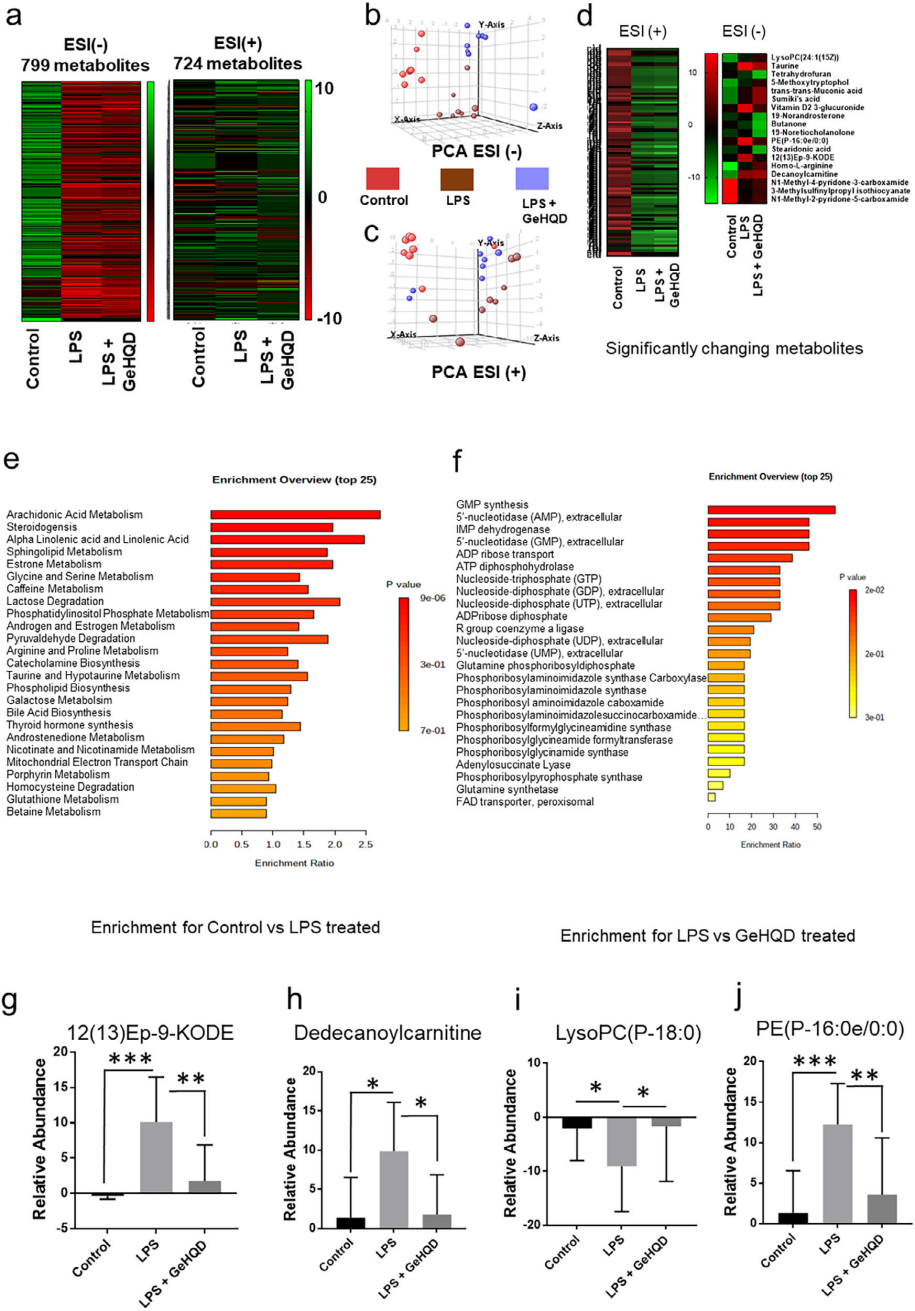
Evaluation of anti‐inflammatory effect of GeHQDs in mice plasma using metabolomics: (a), Heat maps of metabolites detected in both ESI (‐) and ESI (+) modes showing comparative metabolic profile of mice plasma of Control, LPS and LPS+GeHQDs treated mice. (b,c), Principal component analysis (PCA) in different treatment groups showing differences in overall metabolic signature. (d), The panel shows heat maps of all the significantly changing metabolites in respective ESI modes. (e), Enrichment analysis for significantly affected metabolites between the Control and LPS‐treated group. (f), Enrichment analysis for significantly affected metabolites between the LPS and GeHQD‐treated groups. (g–j), Histograms showing comparative abundance of respective metabolites. Eight biological replicates were included per treatment group. The histograms are mean±SD. P‐values were calculated using Two‐way ANOVA with Tukey's multiple comparisons performed for two variables, ^*^ *p* < 0.05. ^**^ *p* < 0.01, ^***^ *p* < 0.001.

Furthermore, to correlate GeHQDs‐induced in vitro cellular metabolic changes with in vivo findings, plasma samples collected from mice in different groups were subjected to metabolomic analysis (using LC‐QTOF/MS). There was a shift in the metabolic profile of LPS‐treated animals toward a pro‐inflammatory phenotype compared to control mice (Figure 8a–e). Enrichment analysis confirmed that there were significant changes in pathways related to arachidonic acid metabolism, steroidogenesis and other lipid metabolism pathways (Figure 8a). Furthermore, PCA analysis demonstrated that treatment with GeHQDs shifted the metabolic profile of LPS mice toward the control group (Figure 8b–c). The enrichment analysis also confirmed significant changes in fatty acid and purine metabolism in GeHQDs‐treated mice (Figure 8f–h; Figure S19a,b). More specifically, in GeHQDs group we observed improvement in the levels of several metabolites which are known as inflammatory markers, such as 12(13)Ep‐9‐KODE, dedecanoylcarnitine, lysophosphatidylcholine (P‐18:0), and phosphoethanolamine (PE) (P‐16:0e/0:0) compared to the control group (Figure 8g–j). These findings confirm that GeHQDs treatment downregulated the LPS‐induced systemic inflammation in a mouse model by shifting the metabolic profile toward anti‐inflammatory phenotype and by upregulating Treg cell number in vivo.

## 3 Discussion

The paradigm of cellular metabolism affecting cell fate and function is globally accepted [43]. It is also known that the phenotype and function of immune cells are highly dependent on cellular metabolism [6]. In recent years, nanotechnology has emerged as a potential tool in designing material‐based strategies for preventing and treating inflammatory and degenerative diseases. In this regard, different forms of synthetic low‐dimensional materials, including 2D nanosheets, 1D nanoparticles, 0D quantum dots, and heterostructures are developed and studied for immunomodulatory applications [12, 13, 14, 33, 49, 50]. These nanomaterials act as immunostimulatory or/and immunosuppressive agents to promote anti‐inflammatory immune responses or tolerance against inflammation through different mechanisms. However, there is very limited data available on the application of nanomaterials in altering the phenotype of immune cells toward an anti‐inflammatory milieu by interacting with metabolic pathways within the immune cells. In the current study we reported for the first time that GeH quantum dots have the potential to shift the metabolic profile of activated T cells toward anti‐inflammatory phenotype by upregulating Treg cell number.

One of the advantages that we witnessed with GeHQDs in the current study was that these nanodots were able to get internalized into the immune cells and have a direct effect by influencing metabolic pathways, which in turn would modulate cell fate and function. We found that, GeHQDs suppressed the levels of pro‐inflammatory cytokines and upregulated anti‐inflammatory cytokines. Further, GeH quantum dots shifted the metabolism of splenocytes toward Tregs under both in vitro and in vivo conditions. The Treg cells express CTLA‐4 and modulate antigen‐presenting cell function. Also, Treg cells secrete anti‐inflammatory cytokines and factors, such as TGF‐β and IL‐10 that promote immunosuppressive responses. In the current study we observed a significant increase in CTLA‐4, TGF‐β, and IL‐10 levels in splenocytes after administering GeHQDs. Another mechanism by which Tregs enforce immune‐suppression is through IL‐2, which is needed for the maintenance of natural Tregs and the induction of newly generated Tregs from T cells. We detected significant upregulation in IL‐2 levels along with downregulation in C‐Jun, IFN‐γ, and TNF‐α levels suggesting the promotion of immunosuppressive function. Furthermore, the metabolic activities in pro‐inflammatory immune cells such as effector T cells and macrophages are supported mostly by glycolytic pathways and the anti‐inflammatory cells including Tregs rely on oxidative phosphorylation (OXPHOS) as well as fatty acid oxidation (FAO) [44]. Also the inhibition of glycolysis promotes Treg phenotype [6, 41]. In the current study metabolomic analysis suggested a significant shift in the overall metabolic environment in T cells after administering GeHQDs. There was a significant downregulation in glycolytic flux following GeHQDs administration as seen by a decrease in abundance of metabolites such as glucose‐6‐phosphate (G6P) and glyceraldehyde‐3‐posphate (G3P) [45]. The G3P feeds into the pentose phosphate pathway (PPP) and a decrease in the abundance of such glycolytic metabolites limits this pathway as well. Classically, PPP flux increases during inflammation [8]. Both glycolysis and PPP consume NAD^+^ and limiting these pathways would partly explain an increase in the abundance of cellular NAD^+^ levels that we observed in the current study after GeHQDs administration. NAD^+^ is previously studied in inflammation and metabolic reprogramming of immune cells, where depletion of cellular NAD^+^ is directly linked to increased inflammation [51]. Further, we also observed an increase in tryptophan breakdown as seen by the increased abundance of acetyl‐N‐formyl‐5‐methoxykynurenamine, which is a part of NAD^+^ biosynthesis pathway [45]. Increase in NAD^+^ favours fatty acid oxidation which can be seen by the low abundance of acylcarnitines in the GeHQDs‐treated group. Increase in NAD^+^ abundance also upregulated the rate of TCA cycle which feeds into the mitochondrial electron transport chain or OXPHOS for energy production [8, 44, 45]. We observed a significant rise in mitochondrial respiration in the GeHQDs‐treated group, which is essential for the immunosuppressive function of Treg cells [52]. It has also been reported that a decrease in amino acid metabolism enhances conversion of naïve T cells toward Tregs and augments their function [53]. Tregs also express catabolic enzymes that can deplete amino acids such as tryptophan and arginine which further help their generation and function [54]. Further, a downregulation in arachidonic acid metabolism is known to stabilize and enhance Treg cell function and phenotype due to a reduction in pro‐inflammatory metabolites [55]. Similarly, a decrease in sphingolipid metabolism enhances Treg phenotype and function by reducing glycolysis through reduced mTORC1 activity and increasing FOXP3 expression [56]. These metabolic changes in response to GeHQDs, together create a cellular environment favoring regulatory T cell phenotype and function. Furthermore, in the current study there was an increase in the abundance of several other key metabolites such as steroid hormones that are reported to augment Treg cell phenotype [41, 44]. The glucocorticoids promote Treg function and proliferation. It is also known that steroid hormones amplify IL‐2‐dependent expansion of Treg cells [57]. Further, the vitamins play an important role in controlling Treg cell proliferation and survival, e.g. retinoic acid (RA) and its derivatives stabilize FOXP3 expression and resist any change in Treg cell phenotype and proliferation [58, 59, 60]. Earlier reports suggest that retinoic acid along with TGF‐β influence induction of Tregs and suppress systemic inflammation in the colitis model [61]. In the current study we observed that administering GeHQDs led to a significant increase in retinoic acid. These evidences strongly suggest that GeHQDs have unique properties to shift immune cell metabolism toward Treg phenotype and suppress inflammation. This change in the metabolic environment by GeHQDs cause phenotypic and functional alterations in the immune cells which we elucidated in our study by looking at their respective interactions.

Furthermore, our in vitro cell culture findings in the current study were validated in a mouse model of LPS‐induced inflammation. We observed a significant upregulation of CD4^+^CD25^+^CD127^lo^ Treg population in GeHQD‐treated LPS mice. The LPS mouse model has been previously well characterized to induce significant transcriptomic perturbations across the peripheral immune cell populations within 4 h of LPS injection. It is observed in mice and pig models that inflammatory kinetics in response to LPS starts at 30 min and peaks at 2–4 h time points, where core transcriptional programs including growth & differentiation, cell signaling, and cytoskeletal‐related genes get perturbed. [62, 63]. We found that GeHQDs through the bloodstream were able to distribute within the major organs including the liver, lungs, and kidneys with no toxic effects. Previously, it has been reported that the application of GeH nanosheets was safe without any signs of toxicity to major organs in CD‐1 mice when administered for a period of 28 days [64]. We also observed a downregulation in the levels of several prominent pro‐inflammatory metabolites in mice plasma after GeHQDs administration suggesting that GeHQDs have a preventing effect toward inflammation. Further, it is very well established that metabolic changes that regulate cell fate and function precede functional changes or occur at the same time. [43, 65] However, to have a greater insight into the mechanism of how GeHQDs regulate metabolic cues, future studies should be performed by inhibiting or activating key metabolic pathways.

There are many reports that state that nanomaterials can alter immune responses by reprogramming immune cells [66, 67]. Also, there is ample evidence available in the literature that suggests that metabolism plays an essential role in directing these immune responses. However, there is a significant gap in the knowledge on how nanomaterials can alter intracellular metabolism and local environment to direct immunomodulation. Here, we have demonstrated that GeHQDs can influence key metabolic pathways of immune cells under in vitro as well as in vivo conditions to trigger Treg cell phenotype and promote immune suppression. We believe this study would open a new paradigm in targeting inflammatory diseases by modulating immune cell metabolism using next‐generation novel nanomaterials.

## 4 Materials and Methods

### 4.1 Conversion of Germanane Nanosheets to GeHQDs

GeH quantum dots were prepared by sonicating 2D GeH nanosheets in an ice bath (# 906026, Sigma Aldrich, Canada). Briefly, 0.1 gram of GeH flakes was gently dispersed in 100 mL of isopropyl alcohol solution and then magnetically stirred for 1 h to expand the interlayer space of nanosheets and break them to smaller pieces. The mixture was sonicated at 4°C for 24 h. The dispersion solution was then centrifuged at 3500 rpm for 30 min and thoroughly washed with ultrapure distilled water to obtain GeH particles in the supernatant. The aqueous suspension of GeH particles was further sonicated at 4°C for 2 weeks until a desired size of GeHQDs was obtained. The colloidal solution was subsequently sterilized by autoclaving at 121°C for 30 min and stored at 4°C for further characterization and experiments.

### 4.2 Morphology and Microstructural Characterization

The microstructural properties of the material were characterized using scanning electron microscopy (FEI Nova, NanoSEM 450 ThermoFisher Scientific Co.), transmission electron microscopy (FEI Talos, F200X S/TEM, ThermoFisher Scientific Co.) at the Manitoba Institute of Materials (MIM), the University of Manitoba, Canada. The surface chemistry and composition of GeH flakes and quantum dots were measured using X‐ray photoelectron spectroscopy (XPS, Kratos Axis Ultra, PHI Quantera Physical Electronics Incorporation). The X‐ray diffraction test of the material was performed by an XRD machine at the University of Alberta, Canada (X‐ray: Cu / 40 kV / 44 mA Goniometer: Ultima IV In‐plane) at 2‐theta 5–80° using a continuous scan with a speed rate of 2.00 degree/min. Brightfield and phase contrast microscopy were performed using Cytation 5 Imaging Multi‐mode Reader (BioTek Instruments).

### 4.3 Particle Size Distribution Measurements by Dynamic Light Scattering (DLS) Analysis

The particle size distribution of GeHQDs suspension was measured in deionized water and cell culture media (RPMI‐1640) using DLS analysis (Litesizer DLS 700 and Kalliope software). The size distribution data were acquired for GeHQDs at a concentration of 100 µg/mL in respective aqueous dispersions. The DLS data was plotted as % size distribution using the number weighted function on the *y*‐axis and particle diameter (nm) on the *x*‐axis respectively.

### 4.4 Biocompatibility of GeHQDs

The biocompatibility of GeHQDs was assessed by incubating the quantum dots with splenocytes isolated from male CD‐1 mice (∼40 g) for a period of 72 h. Briefly, splenocytes were plated on 96 well plate (50 000 cells/well), and treated with GeHQD at two different concentrations 2 and 100 µg/mL for a period of 72 h. The biocompatibility was measured using the lactate dehydrogenase (LDH) cytotoxicity detection kit (Takara Bio Inc. # MK401) using the manufacturer's protocol as described previously [68]. The LDH activity in different groups in the assay plate was measured using Cytation 5 Imaging Multi‐mode Reader (BioTek Instruments). The biocompatibility was also assessed by AnnexinV/PI labelling using flow cytometry (ThermoFisher Scientific # V13242) as per the manufacturer's protocol. The sample acquisition was performed on a Cytoflex flow cytometer (Beckman Coulter) and analyzed using CytExpert software 2.3.1.22 (Beckman Coulter, Brea, California).

### 4.5 In Vitro Immunomodulation Protocol

The splenocytes were isolated from male CD‐1 mice using Histopaque (Sigma–aldrich # 10831) as per the manufacturer's protocol [69]. To assess the immunomodulatory properties of GeHQDs, mouse splenocytes were activated using phytohemagglutinin (PHA). Briefly, splenocytes were suspended in 500 µL of Advanced RPMI complete media (Gibco # 12633012) in a 24‐well plate (4 × 10^6^ cells/well) with 15 µg/mL PHA and GeHQDs (at two different doses 2 and 100 µg/mL) for a period of 72 h at 37°C and 5% CO_2_. The immunomodulatory properties of GeHQDs were assessed by measuring immune cell proliferation, cytokine levels (Biolegend LEGENDplex assay kits, # 740826), gene expression analysis, Treg cell number (flow cytometry), metabolomic analysis and by Seahorse analysis as described in the following sections.

The cell proliferation was measured by using the WST cell proliferation kit and by following the manufacturer's protocol (Abcam # Ab65475) as described previously [70]. The GeHQDs incubated with splenocytes were also assessed for cellular uptake at two different doses, 2 and 100 µg/ml by using A Nikon Ti‐2E microscope.

### 4.6 Flow Cytometry Analysis

The activated mice splenocytes treated with GeHQDs were evaluated for Treg cell number using flow cytometry. Live cells were selected with eFluor 520 (Thermo Fisher Scientific # 65086714) or with CFSE for surface staining (Biolegend # 423801) as per the manufacturer's protocol. Briefly, the cells were washed twice with flow cytometry staining medium (1% bovine serum albumin, 2 mm EDTA and 0.1% sodium azide in PBS). The cells were then stained with surface markers CD4 (PerCP) and CD25 (PE) (Biolegend # 100537 and 162103) at 4°C for 1 h in the dark. They were then fixed with fixation and permeabilization buffer (eBiosciences, Thermo Fisher Scientific # 00‐5523‐00) as per the manufacturer's protocol. The staining for FOXP3 was performed at room temperature by incubating the cells with the antibody (AF647, Biolegend # 320014) for 1 h. Before acquisition, the samples were washed twice with permeabilization buffer and resuspended in flow cytometry staining media. The sample acquisition was done on Cytoflex flow cytometer (Beckman Coulter) with appropriate isotype (Rat IgG2b, κ Isotype Biolegend # 400607 and 400511; Mouse IgG1, κ Isotype Biolegend # 400111) and compensation controls. The compensation was performed using VersaComp Antibody capture bead kit (Beckman Coulter # B22804). The unstained samples (control), isotypes and Fluor minus controls were used for gating. The data was analyzed using CytExpert software 2.3.1.22 (Beckman Coulter, Brea, California) and Flow jo 10.1.0.0 (Beckman Dickinson, Oregon). The stained cells in each group were also cytospun on slides using Statspin Cytofuge System (Iris Sample Processing) at 800 rpm for 4 min and were imaged using Nikon Ti‐2E microscope.

### 4.7 Seahorse Analysis

Mitochondrial function and cellular metabolism in live cells were measured using the Agilent Seahorse XFe24 analyzer. The 24‐well Seahorse plates were coated with cell‐tak (22.5 µg/mL) in 0.1 M Na_2_HCO_3_ solution at pH 8 and room temperature for 2 h. The plates were washed with sterile deionized water and air‐dried. The splenocytes (0.5 × 10^6^ per well) were seeded in XF base and DMEM media containing glucose, L‐glutamine, and sodium pyruvate (Agilent tech # 103334‐100). The cells were incubated at 37°C for 1 h before starting the assay. The adhered cells were washed twice and suspended in 500 µl XF base medium. The baseline oxygen consumption rate (OCR), proton leak, maximal respiration, and non‐mitochondrial respiration were measured sequentially using 1 µm of oligomycin, FCCP (2 µm), 1 µm rotenone, and 1 µm antimycin A as described in previous studies [71]. The data are presented as pmol of oxygen/minute/cell count.

### 4.8 Gene Expression Analysis Using qPCR

The total RNA was extracted using a commercial kit (BIO‐RAD # 7326820) and converted to cDNA using iScript gDNA Clear cDNA Synthesis Kit (BIORAD # 1725035) as per the manufacturer's protocol. The SYBR Green Supermix (BIO‐RAD # 1725271) was used for performing qPCR. The BIO‐RAD C‐1000 Touch real‐time PCR system was used to carry out qRT‐PCR reactions. The data was analyzed using 2^−ΔΔCt^ method to calculate fold change, β‐actin served as the endogenous control. The sequences of primers used for PCR are provided in Table S1.

### 4.9 Metabolomics Analysis

#### 4.9.1 Metabolite Extraction from Cells

The cells in different groups were suspended in 1 mL of ice‐cold methanol (80%) and vortexed at 3000 rpm for 5 min. The samples were sonicated in ice‐cold water bath for 15 min, followed by centrifugation at 12 000 rcf at 4°C, for 15 min. The supernatants were transferred into a clean 2 mL tube and dried under a gentle flow of nitrogen gas. The dry residues were reconstituted in a mixture of methanol: water (4:1, v/v).

#### 4.9.2 Metabolite Extraction from Plasma

Plasma was prepared from the blood collected from mice in heparinized vacutainer tubes. Next, 200 µL of ice‐cold acetonitrile was added to 100 µL of mice plasma and vortexed at 3000 rpm for 1 min followed by 10 min incubation on ice. The mixture was centrifuged at 10 000 RCF for 10 min at 4°C. The supernatants were transferred to a clean eppendorf tube and dried under gentle flow of nitrogen gas. The dried residues were reconstituted in 100 µL of acetonitrile: water (4:1 v/v).

The extracts from cells and plasma containing metabolites were analyzed using Rapid Resolution HPLC system (1290 Infinity Agilent Ltd., Santa Clara CA), coupled with a 6538 UHD Accurate LC‐QTOF‐MS (Agilent Technologies, Santa Clara, CA, USA) containing a Dual electro‐spray ionization (ESI) source. The metabolites were separated using a ZORBAX Extend‐C18 column 2.1 × 50 mm, 1.8um, 600 bar (Agilent Technologies) at 55°C. The samples were run in both ESI positive and negative modes. Data were processed using Agilent Mass Hunter Qualitative analysis software (MHQ, Version B.07). The METLIN database was used to annotate the metabolites, the data collected were then fed into the Metaboanalyst online tool for enrichment and pathway analysis. The heat maps and histograms were generated using Graph pad Prism 10 (San Diego, CA). The data was normalized using total protein for both cells and plasma for consistency and accuracy of measurements. The pooled samples, standards, and calibrants were used as part of our protocol to monitor the accuracy and consistency of results over time.

### 4.10 Polarization of CD4^+^ T Cells Toward Tregs

The CD4^+^ T cells were isolated using Mouse MojoSort CD4^+^ T‐cell Isolation Kit as per the manufacturer's protocol (Biolegend # 480005). The 12‐well plates were aseptically coated with anti‐mouse CD3ε, clone 145‐2C11 (3 µg/mL) (Biolegend # 100339) in PBS and incubated for 2 h at 37°C. The plates were washed three times with PBS, and 1 × 10^6^ CD4^+^ T cells were plated in each well and cultured for 5 days at 37°C, 5% CO_2_, with anti‐mouse CD28, clone 37.51 (3 µg/mL) (Biolegend # 102116), recombinant mouse IL‐2 (5 ng/mL, Biolegend # 575402) and recombinant human TGF‐β (5 ng/mL, Biolegend # 781802) [72]. The cells were analyzed with flow cytometry as described above. Effect of GeHQDs on Treg polarization was observed similarly by stimulating cells with 2 and 100 µg/mL GeHQDs respectively.

### 4.11 T Cell Suppression Assay

The CD4^+^ T cells were stained with 8 µM CFSE stain (ThermoFisher Scientific # 65085084) as per the manufacturer's instructions and resuspended (5 × 10^5^/mL) in RPMI1640 plus media containing 10% FBS, 50 µM β‐mercaptoethanol, 1 mM sodium pyruvate, P/S, MEM Non‐Essential Amino Acids and anti‐mouse CD28 (3 µg/mL). The polarized Tregs and CFSE‐labeled CD4^+^ T cells were co‐cultured in 96‐well tissue culture round‐bottom plates (coated with anti‐mouse CD3ε) at a ratio of 1:4, 1:16, and 1:64 respectively with a decreasing number of Tregs in the presence of GeHQDs (2 and 100 µg/mL) vs control. The cells were incubated at 37°C for 72 h and then the CFSE signal was read using a Cytoflex flow cytometer (Beckman Coulter) [73].

### 4.12 Dendritic Cell Isolation and Coculture with Tregs

Dendritic cells (DCs) were isolated from the femur bone marrow of male CD‐1 mice. Briefly, bone marrow was washed with PBS and after red blood cell lysis, remaining cells were washed with R10 media (RPMI 1640 with Glutamax, 10% FBS, ThermoFisher Scientific), β‐mercaptoethanol (50 µm), and recombinant GM‐CSF (20 ng/mL, Cayman). The non‐adherent DCs were obtained after 7 days of culture and activated overnight with IL‐4 (5 ng/mL) and LPS (1 µg/mL) and washed with PBS. The DCs were then exposed to 323–339 OVA peptide (10 µg/mL) for 1 h at 37°C [74, 75]. The DCs (10 × 10^5^) were cocultured with Tregs (10 × 10^5^) in 24‐well tissue culture plates (coated with anti‐mouse CD3ε) in RPMI plus media with IL‐2 (100IU), GMCSF (10 ng/mL), and LPS (0.1 µg/mL) for a period of 24 h in the presence of GeHQDs (2 and 100 µg/mL) vs. control. The interaction between these cells was analyzed by flow cytometry using anti‐CTLA‐4 and anti‐CD86 antibodies (Biolegend # 106309, 105025), and CFSE staining was performed for cell viability [48, 76].

### 4.13 Effect of GeHQDs of Naïve CD4^+^ T Cells

The naïve CD4^+^ T cells were harvested using MojoSort Mouse CD4^+^ Naïve T Cell Isolation Kit (Biolegend # 480039) from spleen and axillary & inguinal lymph nodes of male CD‐1 mice as per the manufacturer's protocol. The effect of GeHQDs on cell proliferation was analyzed using the WST cell proliferation kit (Abcam # Ab65475) as per the manufacturer's instructions. Similarly, mitochondrial membrane potential was assessed using TMRE assay (Invitrogen # T669) as per the manufacturer's protocol. The number of Treg cells was analyzed using flow cytometry, and TGF‐β as well as TNF‐α levels were measured as described in the following sections.

### 4.14 Ethics and Animal Protocol

The animal protocol 21‐002 (AC11655) used in this study was approved by the University of Manitoba Animal Care Committee. The study followed all the guidelines set by the Canadian Council on Animal Care. Male CD‐1 mice (∼40 g) were used to isolate splenocytes for in vitro studies and for in vivo LPS‐induced inflammation model. All procedures were performed at R.O. Burrell laboratory at the St. Boniface Hospital Research Centre, University of Manitoba, Winnipeg as per the standard operating guidelines.

### 4.15 In Vivo Mouse Model and Immunomodulation

A mouse model of systemic inflammation was created in CD‐1 mice. The animals were divided into 3 groups viz. control, LPS‐treated and LPS+GeHQD treatment. The experimental animals received an intraperitoneal injection of LPS at a dose of 1 mg/kg body wt. The dose and treatment protocol were chosen based on the existing literature. The animals in GeHQD group received an intraperitoneal injection of 2 mg/kg body wt of GeH quantum dots along with LPS. After 4 h of LPS injection and GeHQDs treatment, peritoneal cells (through intraperitoneal lavage in PBS containing 5 mm EDTA) and blood samples (through the jugular vein) were collected. The cells were labelled with CFSE. The neutrophil cell number in peritoneal fluid was measured by counting the number of CD11b^+^Ly6G^+^ (Biolegend # CD11b‐PE 101207 and Ly6G‐APC 127613) cells using flow cytometry as described above. The Treg cell number was measured in blood (plasma) samples by counting the number of CD4^+^CD25^+^CD127^lo^ (CD4‐PerCP # 100537; CD25‐PE # 162103; CD127‐APC # 135011) cells using flow cytometry [77, 78, 79]. The plasma samples were also used for cytokine array analysis (as described in the following section) and for metabolomics analysis (using the LC‐QTOF/MS system as described above).

The in vivo biocompatibility of GeHQDs was assessed by performing hematoxylin and eosin staining in lung, kidney, and liver tissue sections as described in the following sections.

### 4.16 Cytokine Analysis

The quantitative estimation of cytokines (IFN‐gamma, IL‐6, IL‐17A, sCD40L, IL17A, IL‐4, TNF‐α, IL‐10, and TGF‐β) was performed using Biolegend LEGENDplex multi‐analyte flow assay kits (Biolegend # 740826) by following the manufacturer's instructions. Briefly, the capture beads with respective antibodies were incubated with analytes for 2 h at room temperature on a shaker. The samples were washed twice and detection antibodies (biotinylated) were added and incubated for 1 h at room temperature. Next, streptavidin‐phycoerythrin was added to the wells and incubated for 30 min at room temperature under shaking conditions. The wells were washed twice and read on a Cytoflex flow cytometer (Beckman Coulter). The analysis was performed on Biolegend LEGENDplex data analysis software. The TNF‐α, IL‐10, and TGF‐β levels were analyzed using ELISA kits and following the manufacturer's protocol (Biolegend # 430904, 431414, and 433007), and normalized against total viable cells using Nucblue viability stain as secreted cytokines are a function of viability (Thermo Fisher Scientific # R37605).

### 4.17 GeHQD Biodistribution

The GeHQDs when excited by a strong laser exhibit detectable fluorescence at the Cy5‐647 nm channel. To determine the biodistribution of GeHQDs after injecting them into the mice, the spleen, lung, liver, and kidney were collected and fixed in 10% formalin at room temperature, which were then embedded in paraffin to produce 5 µm thick sections mounted on glass slides. The paraffin was removed with xylene followed by rehydration with ethanol. The sections were mounted with Prolong Diamond Antifade Mountant containing DAPI (# P36971, Thermo Fisher Scientific) and imaged using a Nikon Ti‐2E fluorescence microscope using NiS element software [12].

### 4.18 Hematoxylin and Eosin Staining

The in vivo biocompatibility of GeHQDs was assessed by performing hematoxylin and eosin (H and E staining) staining in lung, kidney, liver, and spleen tissue sections. The paraffin wax was removed from the sections in xylene, followed by hydrating the sections and staining with hematoxylin and eosin using a standard protocol [12].

### 4.19 Statistical Analysis

All of the flow cytometry, in vitro cytokine analysis and Seahorse assays were normalized to viable cell counts. Metabolomics readouts were normalized to total protein content prior to statistical analysis. The data were represented as mean ±SD unless otherwise indicated in the Figure legends. The sample sizes were *n* = 4–6 for in vitro assays and *n* = 5–10 for in vivo assays unless otherwise stated in the legends of respective Figures. One‐way analysis of variance (ANOVA) with Tukey's honest multiple comparison test was applied for three or more groups with one variable. The two‐way analysis of variance with Tukey's multiple comparisons was performed for two variables. The *p*‐value with <0.05 was considered significant. Throughout the manuscript ^*^ indicates *p* < 0.05; ^**^ indicate *p* < 0.01; ^***^ indicate *p* < 0.001; ^****^ indicate *p* < 0.0001 and ns for *p* > 0.05. The statistical analysis was performed using Prism version 10.2.2 for windows (Graphpad, San Diego, California). Biorender was used to design some of the Figure panels.

## Author Contributions

This study was conceptualized and designed by A.S., A.R., S.D. A.S., A.R., W.Y., K.N.A., L.R.S., and M.A. carried out the experiments and acquired the data. A.S., A.R., M.A., Q.G., and S.D. interpreted the data and performed statistical and formal analysis. A.S., A.R., and S.D. designed the Figures and drafted the manuscript. All authors read and approved the final manuscript.

## Conflicts of Interest

The authors declare no conflicts of interest.

## Supporting information




**Supporting File**: adfm74031‐sup‐0001‐SuppMat.docx.

## Data Availability

The data that support the findings of this study are available from the corresponding author upon reasonable request.
