# Supplementary material for: Germanane Quantum Dots Promote Metabolic Reprogramming of Immune Cells Toward Regulatory T Cells and Suppress Inflammation In Vitro and In Vivo
Source: Adv Funct Mater. 2026 Jan 18;36(34):e21057. doi: 10.1002/adfm.202521057 (PMC13494919; doi:10.1002/adfm.202521057)
Supplement: Supplementary file 1 [file ADFM-36-e21057-s001.docx]

**Supplementary Information**

**Germanane quantum dots promote metabolic reprogramming of immune cells towards regulatory T cells and suppress inflammation *in vitro* and *in vivo***

Abhay Srivastava^a,^**^†^**, Alireza Rafieerad^a,^**^†^**, Weiang Yan^a^, Keshav Narayan Alagarsamy^a^, Qingdong Guan^c^, Leena Regi Saleth^a^, Michael Aliani^b^, Sanjiv Dhingra^a,^**^*^**

*^a^Institute of Cardiovascular Sciences, St. Boniface Hospital Albrechtsen Research Centre, Department of Physiology and Pathophysiology, Rady Faculty of Health Sciences, Biomedical Engineering Program, University of Manitoba, Winnipeg, Manitoba, R3E 0W2, Canada*

*^b^Division of Neurodegenerative Disorders, St. Boniface General Hospital Albrechtsen Research Centre, University of Manitoba, Winnipeg, Canada*

*^c^Department of Immunology and Internal Medicine, Manitoba Blood and Marrow Transplant Program, CancerCare Manitoba; University of Manitoba, Winnipeg, Canada.*

**^†^ *These authors are co-first authors and contributed equally to this work.***

***Correspondence:**

**Sanjiv Dhingra, PhD, FAHA, FIACS, FAPS**

Professor

Institute of Cardiovascular Sciences

St. Boniface Hospital Albrechtsen Research Centre

Department of Physiology and Pathophysiology

Rady Faculty of Health Sciences, Max Rady College of Medicine

Biomedical Engineering Program

University of Manitoba

R-3028-2, 351 Tache Avenue, Winnipeg, R2H2A6, Canada

Email: [sdhingra@sbrc.ca](mailto:sdhingra@sbrc.ca)

**Supplementary Fig. S1** Microstructure characterization of GeHQDs: TEM image showing distribution of GeHQDs into germanane nanosheets. High-resolution TEM images of GeH nanosheets and derived quantum dots.

**Supplementary Fig. S2:** The particle size distribution measurement of GeHQDs in deionized water (blue plot) and RPMI-1640 cell culture media (red plot) using DLS.

**Supplementary Fig. S****3**: The XPS wide-scan spectra of GeH flakes and after conversion into GeHQDs. The XPS sample preparation was performed at normal air atmosphere and room temperature, explaining the possibility of surface air oxidation or carbon dioxide contamination.

**Supplementary Fig. S4:** Optical absorption characterization of GeHQDs: Phase contrast (a, b) and bright field (c, d) microscopy of GeHQDs at day 60 of synthesis. These data confirm the long-term stability and dispersibility of quantum dots. N=4.

**Supplementary Fig. S5** GeHQDs are biocompatible and do not cause cytotoxicity: **a,** Splenocytes were treated with 2, 10, 25, 50 and 100 µg/ml of GeHQDs for 72 hr. The cytotoxicity was assessed by measuring LDH release from the splenocytes after GeHQDs treatment. **b**, Splenocytes were analysed using Annexin PI staining and flow cytometry. Four biological replicates were included per treatment group. The histograms are mean±SD. P-values were calculated using One-way ANOVA with Tukey’s honest multiple comparison test, *P<0.05. **P<0.01, *** P<0.001 and ns P>0.05.

**Supplementary Fig. S6 a-d,** The bright field images of splenocytes. Exposure to PHA (b) led to increase in the proliferation and aggregation of cells. The GeHQD treatment (c,d) at two different doses, 2 µg/ml and 100 µg/ml downregulated the proliferation of splenocytes, and there was reduced aggregation of cells. N=4.

**Supplementary Fig. S7** Effect of GeHQDs on pro inflammatory and anti-inflammatory cytokines: **a-l**, Histograms showing comparative gene expression of IL-10, TNFα, IFNγ, IL2, CTLA4, CBF beta, TbX21, IRF4, STAT3, IDO1, Granzyme A and C-Jun respectively. Four biological replicates were included per treatment group. The histograms are mean±SD. P-values were calculated using One-way ANOVA with Tukey’s honest multiple comparison test, *P<0.05. **P<0.01 and *** P<0.001.

**Supplementary Fig. S8** Panel shows representative images of CD25^+^FoxP3^+^ Treg cells in respective groups using fluorescent microscopy.

**Supplementary Fig. S9 a-d,** Gating strategy for Treg cell population analysis. **e,** flow cytometric analysis of Treg cell population.

**Supplementary Fig. S10 A-B**, Pathway analysis for significantly affected metabolites between the PHA and GeHQD treated groups.

**Supplementary Fig. S11** **a**, Confirmation of Treg cell polarization. **b-d**, Panels show Treg cell suppression cocultured at different ratios with CD4^+^ T cells.

**Supplementary Fig. S12** **a-e** Gating strategy for CD86^+^CTLA4^+^ Treg cell population and CD86^+^ DCs respectively.

**Supplementary Fig. S13 a-h:** Effect of GeHQDs on naïve CD4^+^ T cells: a-d, Flow cytometry analysis for CD4^+^CD25^+^FoxP3^+^ Treg cell phenotype of naïve CD4^+^ T cells when incubated with GeHQDs. e-f, Histograms showing increase in cell proliferation and mitochondrial membrane potential of naïve CD4^+^ T cells when treated with GeHQDs. g-h, Histogram showing quantification of TGF-β and TNF-α secretion when naïve CD4^+^ T cells were treated with GeHQDs. Four biological replicates were included per treatment group. Four biological replicates were included per treatment group. The histograms are mean±SD. P-values were calculated using One-way ANOVA with Tukey’s honest multiple comparison test, *P<0.05. **P<0.01, *** P<0.001

**Supplementary Fig. S14** **a**, Panel showing red fluorescent signal from GeHQDs when excited by a high-powered laser. **b**, Biodistribution of GeHQDs in blood vessels and major organs (liver, lungs, kidney and spleen).

**Supplementary Fig. S15 a-c,** Mice treated with LPS and GeHQDs were scored on acute inflammatory phenotypic responses including temperature, fur appearance and activity. Ten biological replicates were included per treatment group. The histograms are mean±SD. P-values were calculated using One-way ANOVA with Tukey’s honest multiple comparison test, *P<0.05.

**Supplementary Fig. S16 a-e,** Gating strategy for *in vivo* Treg cell population analysis in mice blood.

**Supplementary Fig. S17 a-d,** Gating strategy for *in vivo* granulocyte population analysis in mice intraperitoneal lavage.

**Supplementary Fig. S18** Panel a and b show representative images of Treg cells and neutrophils in respective groups using fluorescent microscopy.

**Supplementary Fig. S19 a-e,** Pathway analysis for significantly affected metabolites between the LPS and GeHQDs treated groups.

**Supplementary table S1: The details of primers use for qPCR analysis:**

| **Gene** | **Forward seq (5'->3')** | **Reverse seq (5'->3')** |
| --- | --- | --- |
| Jun/AP1 | TGGGCACATCACCACTACAC | TCTGGCTATGCAGTTCAGCC |
| Cbfb | GACAAACACCTAGCCGGGAA | GGCTCGCTCCTCATCAAACT |
| Tbx21/tbet | TGTTCCCAGCCGTTTCTACC | GCTCGGAACTCCGCTTCATA |
| Irf4 | CAGCCCAGCAGGTTCATAACT | TGGTCAGGGGCATAATCCCT |
| Stat3 | CAGGTAGTGCTGCCCCGTA | ATGGTATTGCTGCAGGTCGT |
| Ido1 | GAGTAGACAGCAATGGCACTCA | TGGTAGAGCAAAGCCCACATC |
| Gzma | TTTTGCGAGGCATCACCTCT | TCAGACACAGAAGTGACAGGG |
| IL-2 | GCCCCAAGGGCTCAAAAATG | GCGCTTACTTTGTGCTGTCC |
| TNF-alpha | TAGCCCACGTCGTAGCAAAC | GCAGCCTTGTCCCTTGAAGA |
| TGF-beta | ACTGGAGTTGTACGGCAGTG | GGGGCTGATCCCGTTGATT |
| IFN-gamma | AGCAAGGCGAAAAAGGATGC | TCATTGAATGCTTGGCGCTG |
| IL-10 | TGAATTCCCTGGGTGAGAAGC | CATTCATGGCCTTGTAGACACC |
| CTLA4 | CCGAGTCTGTGTGGGTTCAA | GGTCCTCAGGGAGCAGAGTA |
| Beta actin | AGGAGTACGATGAGTCCGGC | AAAACGCAGCTCAGTAACAGTC |
